# Beyond lipids: a precision prevention blueprint for China's residual risk

**DOI:** 10.3389/fmed.2026.1929544

**Published:** 2026-08-17

**Authors:** Ming Chi, Taoming Qian, Jirong Zhang, Weiqing Qiao, Juan Jin

**Affiliations:** 1 Graduate School, Heilongjiang University of Chinese Medicine, Harbin, China; 2 Department of Emergency, Longhua Hospital, Shanghai University of Traditional Chinese Medicine, Shanghai, China; 3 Cardiovascular Department, The First Affiliated Hospital of Heilongjiang University of Chinese Medicine, Harbin, China

**Keywords:** Chinese cardiovascular disease, colchicine, high-sensitivity C-reactive protein, lipoprotein(a), precision prevention, residual inflammatory risk, residual risk, secondary prevention

## Abstract

Despite achieving guideline-recommended lipid levels, patients with atherosclerotic cardiovascular disease (ASCVD) retain substantial residual risk arising from heterogeneous biological pathways, including persistent inflammation and genetically determined atherogenic susceptibility. High-sensitivity C-reactive protein (hs-CRP) provides a pragmatic measure of potentially modifiable inflammatory activity, whereas lipoprotein(a) [Lp(a)] identifies relatively stable, genetically mediated risk that is not adequately captured by standard lipid panels. The 2025 American College of Cardiology Scientific Statement highlights hs-CRP as a clinically useful inflammatory-risk marker and supports consideration of low-dose colchicine in appropriately selected secondary-prevention patients. The 2026 ACC/AHA multisociety dyslipidemia guideline further provides a Class I recommendation for once-in-a-lifetime Lp(a) measurement, creating a complementary multimodal framework for residual-risk assessment. In China, where cardiovascular disease accounts for a substantial proportion of deaths and the national cardiovascular burden continues to increase, implementation of this framework remains limited. Routine hs-CRP measurement is uncommon, cardiovascular use of colchicine remains negligible, and Lp(a) testing is still concentrated in selected tertiary centers. Emerging East Asian data and a recent Chinese expert advisory suggest that an hs-CRP threshold of approximately 1.0 mg/L may improve inflammatory-risk discrimination in Chinese patients with coronary artery disease; however, this threshold should not yet be interpreted as a stand-alone treatment threshold for colchicine. In this perspective, we compare international recommendations with current Chinese practice, analyze barriers related to infrastructure, safety, education, standardization, and evidence localization, and propose a multimodal implementation framework. Short-term priorities include once-in-a-lifetime Lp(a) measurement, context-specific hs-CRP assessment, intensified management of modifiable atherosclerotic risk factors, and carefully selected use of low-dose colchicine in secondary prevention. Elevated Lp(a) should prompt comprehensive risk-factor intensification and, when appropriate, family-based evaluation, but should not by itself constitute an indication for colchicine. Medium- and long-term priorities include pragmatic trials, real-world registries, assay standardization, cost-effectiveness studies, and policy initiatives to integrate residual-risk assessment into Chinese cardiovascular prevention pathways.

## Introduction

Despite remarkable advances in lipid-lowering and antithrombotic therapies, atherosclerotic cardiovascular disease (ASCVD) continues to exact a substantial toll. Residual risk after achievement of guideline-recommended low-density lipoprotein cholesterol (LDL-C) levels is biologically heterogeneous and may reflect persistent inflammation, genetically determined atherogenic factors, thrombosis, metabolic dysfunction, and incomplete control of conventional risk factors. High-sensitivity C-reactive protein (hs-CRP) is a widely available biomarker of systemic inflammatory activity and has emerged as an independent predictor of recurrent major adverse cardiovascular events (MACE) (1). Importantly, hs-CRP should be interpreted as a risk marker rather than a direct surrogate for atherosclerotic burden, particularly when acute infection or other inflammatory conditions are present.

The 2025 American College of Cardiology (ACC) Scientific Statement on Inflammation and Cardiovascular Disease highlights the clinical relevance of inflammatory-risk assessment and supports consideration of targeted anti-inflammatory therapy, including low-dose colchicine, in appropriately selected patients with established ASCVD (2). In parallel, the 2026 ACC/AHA multisociety dyslipidemia guideline provides (3) a Class I recommendation for once-in-a-lifetime measurement of lipoprotein(a) [Lp(a)] in adults to identify genetically determined atherogenic risk that is not adequately captured by routine lipid profiles. Lp(a) and hs-CRP should therefore be viewed as complementary rather than interchangeable biomarkers: Lp(a) reflects relatively stable inherited susceptibility, whereas hs-CRP reflects dynamic inflammatory activity that may be influenced by obesity, diabetes, smoking, infection, kidney disease, and other comorbidities. This distinction is clinically important. An elevated Lp(a) concentration should prompt intensification of LDL-C lowering and comprehensive management of modifiable cardiovascular risk factors, consistent with the risk-stratified, LDL-C-centered treatment framework recommended in the 2023 Chinese guideline for lipid management, and may support family-based evaluation when clinically appropriate (4). It should not, however, be considered an independent indication for colchicine. Conversely, persistent hs-CRP elevation may help identify a subgroup in whom anti-inflammatory therapy could be considered, provided that the patient has an appropriate secondary-prevention indication and no major contraindication. Integrating these two biomarkers may improve residual-risk phenotyping, although this combined approach has not yet been validated as a treatment-allocation algorithm.

This renewed global focus on inflammation holds particular urgency for China. The Report on Cardiovascular Health and Diseases in China 2024 paints a stark picture: cardiovascular disease (CVD) is responsible for nearly half of all deaths, with crude mortality rates of 305.39 per 100,000 in urban areas and 364.16 per 100,000 in rural areas in 2021 (5). Furthermore, recent national surveillance data reveal a staggering crude incidence rate of 620.33 per 100,000 for cardiovascular and cerebrovascular events among adults in 2023 (5–7). A comprehensive analysis (8) of the Global Burden of Disease further underscored the escalating challenge, revealing a 140% increase in CVD prevalence and a 53% rise in disability-adjusted life years in China since 1990, with projections indicating a persistently high burden in the coming decades. This immense burden is amplified by a confluence of highly prevalent comorbidities—hypertension, type 2 diabetes, obesity, dyslipidemia, and metabolic dysfunction-associated steatotic liver disease—that synergistically fan systemic inflammation. Consequently, Chinese ASCVD patients may experience substantial residual inflammatory risk because of the high prevalence of metabolic comorbidities and the increasing burden of obesity, diabetes, smoking, and chronic kidney disease. However, direct comparisons of residual inflammatory risk between Chinese and Western populations remain heterogeneous and should not be overgeneralized. Importantly, emerging data suggest that the optimal hs-CRP threshold for risk discrimination in East Asian populations may not be the canonical ≥2 mg/L, with preliminary evidence favoring a lower cut-point such as ≥1.0 mg/L, a nuance with profound implications for local practice (9). A 2026 Chinese expert advisory proposes hs-CRP ≥1 mg/L as a clinically useful threshold for identifying residual inflammatory risk in Chinese patients with coronary artery disease, together with consideration of complementary markers such as the neutrophil-to-lymphocyte ratio (10). This recommendation is relevant for risk stratification, but whether the threshold should directly guide colchicine initiation requires prospective outcome validation.

Despite a robust international evidence base that is rapidly evolving, translation into routine Chinese clinical practice remains profoundly limited. Gaps in screening, awareness, prescribing patterns, and localized evidence generation are substantial and persistent. In this perspective, we systematically dissect these disconnects, contrast global standards with local realities, and advance a pragmatic, evidence-anchored framework for clinicians, researchers, and policymakers. By integrating the 2025 ACC inflammation statement with the 2026 ACC/AHA dyslipidemia guideline, our aim is to develop a clinically realistic framework that combines inherited atherogenic risk, dynamic inflammatory risk, and evidence-based secondary prevention for Chinese patients.

## Global evidence summary

The inflammatory hypothesis of atherothrombosis, long suspected from observational data, was transformed into clinical reality by the CANTOS trial, which demonstrated that specifically targeting the interleukin-1β innate immunity pathway with canakinumab reduced MACE independently of lipid lowering (11). This proof-of-concept provided the intellectual springboard for repositioning the venerable, inexpensive agent colchicine—which inhibits the NLRP3 inflammasome, microtubule polymerization, and neutrophil-platelet aggregates—as a broad-spectrum anti-inflammatory intervention for secondary prevention.

The 2025 ACC statement synthesizes this mechanistic rationale with contemporary clinical-trial data to make a compelling case for routine inflammation assessment and treatment (2). Two landmark trials anchor this recommendation. The LoDoCo2 trial randomized 5,522 patients with chronic coronary disease on guideline-directed therapy to colchicine 0.5 mg daily or placebo; over a median follow-up of 28.6 months, colchicine reduced the primary composite endpoint of cardiovascular death, spontaneous myocardial infarction, ischemic stroke, or ischemia-driven revascularization from 9.6 to 6.8% [hazard ratio (HR) 0.69, 95% confidence interval (CI) 0.57–0.83; *P* < 0.001], a 31% relative risk reduction with a safety profile dominated by mild, self-limited gastrointestinal effects (12). In the COLCOT trial, conducted in 4,745 patients shortly after myocardial infarction, low-dose colchicine achieved a 23% reduction in the primary ischemic endpoint (5.5% vs. 7.1%; HR 0.77, 95% CI 0.61–0.96; *P* = 0.02), driven largely by reductions in stroke and urgent revascularization (13). A subsequent patient-level systematic review and meta-analysis of randomized trials confirmed a consistent, significant benefit of low-dose colchicine on MACE, with no increase in serious infections, cancer, or death (14). Beyond clinical endpoints, the COLOCT randomized clinical trial (15) provided direct mechanistic evidence supporting these benefits. In 128 Chinese patients with acute coronary syndrome, Yu et al. demonstrated that adding colchicine (0.5 mg daily) to standard therapy for 12 months significantly increased minimal fibrous cap thickness (difference, 34.2 μm; *P* = 0.006) and reduced the mean angular extension of macrophages (*P* = 0.044), as assessed by optical coherence tomography. These findings directly link colchicine's anti-inflammatory action to improved coronary plaque stability. These data prompted the 2023 AHA/ACC chronic coronary disease guideline to assign a Class IIb recommendation for low-dose colchicine in secondary prevention, a stance that the 2025 ACC statement further reinforces and amplifies (16).

It is crucial to recognize that residual inflammatory risk is not a rare phenomenon. Across major statin trials and real-world registries, 30%−40% of optimally treated patients maintain hs-CRP concentrations ≥2 mg/L, a phenotype that independently confers a risk comparable to or exceeding that conferred by residual cholesterol risk (1). This high prevalence underscores the scale of the unmet need and the potential population benefit of addressing inflammation alongside lipids.

## Gap analysis in Chinese clinical practice

Chinese ASCVD patients face a double disadvantage in the context of prevalent metabolic syndrome: a disproportionately high inflammatory burden coupled with systematic under-recognition and under-treatment. Multiple domestic cohorts document that residual inflammatory risk is strikingly prevalent. Yu et al., studying Chinese patients with acute coronary syndrome undergoing percutaneous coronary intervention (PCI), found that over 40% exhibited persistently elevated hs-CRP (≥2 mg/L) despite contemporary statin therapy, and that this elevation strongly predicted subsequent MACE (17). The amplification of risk is particularly striking in metabolic comorbidities. Chen et al. reported that among statin-treated patients receiving drug-eluting stents, the combination of diabetes and elevated hs-CRP synergistically multiplied the hazard of adverse events, highlighting a critical high-risk subgroup that is especially common in China (18). Moreover, in a nationwide prospective cohort of middle-aged Chinese adults free from baseline cardiovascular disease, Dong et al. demonstrated that elevated hs-CRP was linearly and independently associated with an increased risk of developing CVD, and that adding hs-CRP to traditional risk models yielded a significant net reclassification improvement of 7.85%, reinforcing the notion that inflammatory risk is not confined to the acute setting (19).

The prognostic importance of residual inflammatory risk extends beyond coronary disease into the cerebrovascular domain. In an analysis of the Third China National Stroke Registry, Liu et al. showed that persistent high RIR (hs-CRP ≥3 mg/L) independently predicted 1-year stroke recurrence among patients with acute ischemic stroke or transient ischemic attack, with an even more pronounced effect in those harboring intracranial artery stenosis or large-artery atherosclerosis subtype (20). Furthermore, Wang et al., leveraging data from CHARLS, HRS, and a Chinese hospital cohort, demonstrated that persistent high RIR strongly predicted incident cardiometabolic multimorbidity—the progression from a single cardiometabolic disease to multiple conditions—across all three populations, after comprehensive adjustment for traditional risk factors (21). These findings collectively illustrate that RIR is a pan-vascular and multisystem threat, not merely a coronary-specific phenomenon.

Critically, the optimal risk-stratification threshold for East Asians appears to differ from Western benchmarks, and this has now been codified. Xiong et al. systematically evaluated hs-CRP cutoff values in East Asian coronary heart disease populations and provided compelling evidence that a lower cutoff—≥1.0 mg/L—offers superior sensitivity and net reclassification improvement compared with the universally applied 2 mg/L (9). The 2026 Chinese expert advisory formally adopts this evidence, recommending hs-CRP ≥1 mg/L as the standard for residual inflammatory risk assessment in Chinese patients, together with neutrophil-to-lymphocyte ratio evaluation, and proposes a dual-biomarker risk stratification scheme that categorizes patients into low, intermediate, and high inflammatory risk (10). Concurrently, emerging metabolomic data are enriching the biomarker landscape: Zhang et al. found that specific plasma ceramides—most notably Cer(d18:1/16:0)—were independently associated with high RIR in Chinese patients with coronary artery disease, suggesting that lipidomic profiling may add granularity to inflammation phenotyping beyond hs-CRP alone (22).

The clinical impact of RIR extends beyond risk prediction to therapeutic decision-making. A prospective observational study from Fuwai Hospital demonstrated that among post-PCI patients with high RIR (hs-CRP ≥2 mg/L), prolonging dual antiplatelet therapy beyond 12 months significantly reduced the composite of all-cause death, myocardial infarction, stent thrombosis, or stroke (1.5% vs. 4.2%; adjusted HR 0.35, 95% CI 0.22–0.54) without increasing clinically significant bleeding (23). This finding implies that inflammatory status could be leveraged to individualize antithrombotic strategies—a proposition that awaits prospective validation.

Despite these data, routine hs-CRP measurement is conspicuously absent from most Chinese cardiology and primary care settings, particularly outside tertiary hospitals. The 2024 primary-care version of the Chinese guideline for lipid management (24) provides a practical LDL-C-centered framework for risk assessment, treatment initiation, follow-up, and referral in primary-care settings. This framework could serve as the implementation foundation for selectively incorporating Lp(a) and hs-CRP assessment into primary-care and referral pathways. Consequently, colchicine use for ASCVD secondary prevention remains negligible, with prescribing almost entirely confined to traditional rheumatologic or pericardial indications. A recent comprehensive meta-analysis reaffirmed the efficacy of low-dose colchicine in reducing major adverse cardiovascular events across the spectrum of coronary artery disease (25). However, these and other pivotal trials have included few or no Chinese participants, leaving critical evidence void regarding generalizability to our local populations. Several barriers converge to produce this gap. *Accessibility and infrastructure*: though hs-CRP assays are inexpensive, their integration into standard acute coronary syndrome and chronic disease management pathways is limited by equipment shortages, insufficient technician training, and lack of electronic health record prompts. *Safety and tolerability concerns*: colchicine's narrow therapeutic window, its gastrointestinal side effects, and its interactions with CYP3A4 and P-glycoprotein substrates—including certain statins and calcium-channel blockers widely used in China—breed understandable clinician caution (26). *Cognitive and educational inertia*: many physicians remain anchored in a dual lipid-thrombosis paradigm, viewing inflammation as a secondary phenomenon rather than an independent, modifiable therapeutic target. *Evidence localization deficit*: large-scale Chinese randomized controlled trials and comprehensive real-world registries evaluating the efficacy, safety, cost-effectiveness, and optimal hs-CRP cutoffs for colchicine therapy are lacking, perpetuating uncertainty and guideline ambiguity.

### A parallel implementation gap concerns Lp(a)

Despite the Class I recommendation for once-in-a-lifetime measurement, Lp(a) testing remains inconsistently available across Chinese hospitals and is rarely embedded in routine ASCVD, lipid-clinic, or primary-care workflows. Barriers include limited clinician awareness, variable assay availability, inconsistent reporting in mg/dL or nmol/L, uncertainty regarding the interpretation of apo(a) isoform-related assay variability, and the absence of clear downstream management pathways. In addition, an elevated Lp(a) result is often treated as an isolated laboratory abnormality rather than as a signal to intensify LDL-C lowering, assess premature ASCVD in first-degree relatives, and optimize global risk-factor control.

The biological differences between Lp(a) and hs-CRP also have important implementation implications. Lp(a) is predominantly genetically determined and generally requires only one measurement during adulthood, whereas hs-CRP is a dynamic biomarker that should be measured in a clinically stable state and repeated when an unexpected elevation may reflect acute infection or another transient condition. These biomarkers should therefore be incorporated into different but complementary clinical pathways rather than treated as interchangeable components of a single laboratory panel.

**Figure 1:**
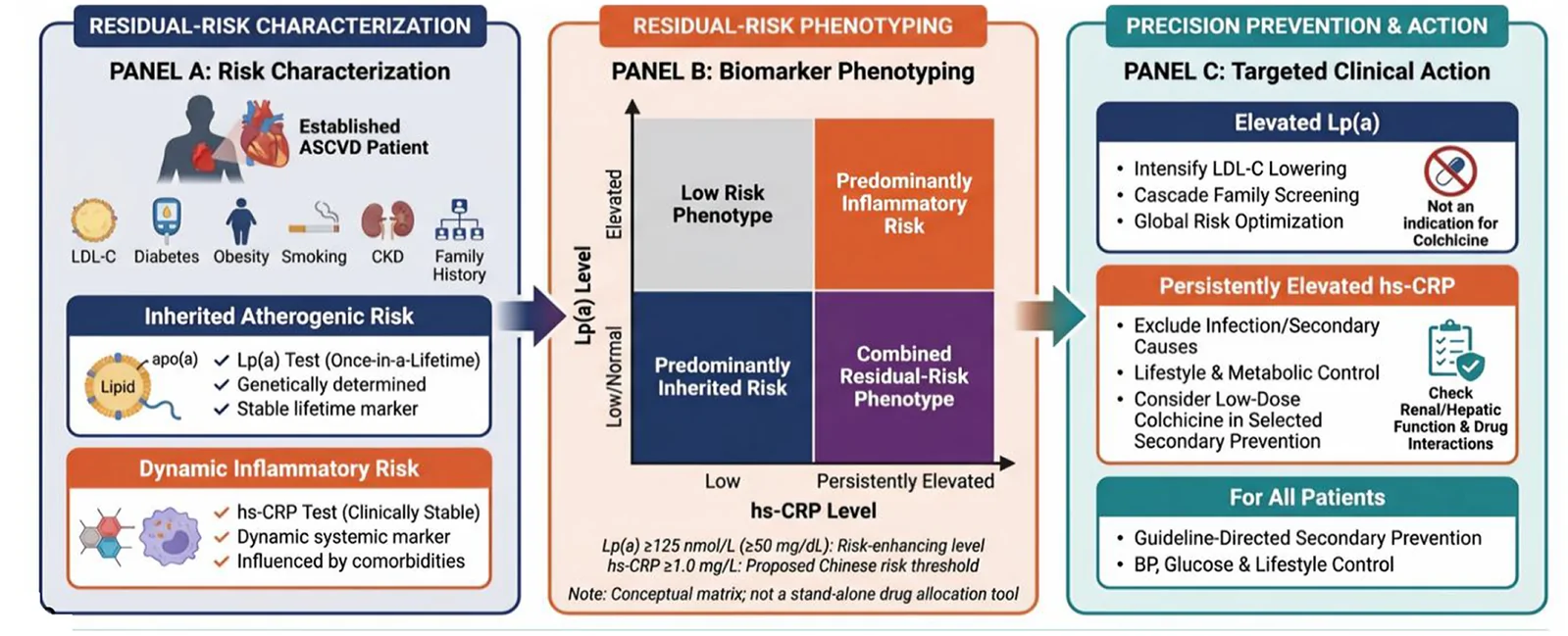
An integrated multimodal framework for residual-risk assessment and precision prevention in Chinese ASCVD. **(A)** Residual-risk characterization in patients with established ASCVD, distinguishing once-in-a-lifetime Lp(a) measurement (relatively stable, genetically determined atherogenic risk) from context-specific hs-CRP assessment performed in the clinically stable state (dynamic inflammatory risk influenced by metabolic comorbidities). **(B)** Conceptual 2 × 2 biomarker phenotyping matrix integrating Lp(a) and hs-CRP levels to identify predominantly inherited risk, predominantly inflammatory risk, or a combined residual-risk phenotype. Thresholds shown are Lp(a) ≥ 125 nmol/L (≥50 mg/dL) as a commonly used risk-enhancing level and hs-CRP ≥1.0 mg/L as a proposed Chinese risk-stratification threshold. The matrix is conceptual and has not been validated as a stand-alone treatment-allocation tool. **(C)** Targeted clinical actions stratified by biomarker phenotype: elevated Lp(a) prompts intensified LDL-C lowering, cascade family evaluation when appropriate, and global risk-factor optimization, but is not an independent indication for colchicine; persistent hs-CRP elevation supports exclusion of secondary causes, lifestyle and metabolic control, and consideration of low-dose colchicine in carefully selected secondary-prevention patients after safety assessment.

These multilayered barriers perpetuate preventable recurrent events and underscore an urgent imperative for context-sensitive adaptation. Figure 1 presents an integrated multimodal framework that distinguishes inherited atherogenic risk [Lp(a)] from dynamic inflammatory risk (hs-CRP) and outlines corresponding precision-prevention actions for Chinese patients with ASCVD.

### Solutions and authors' perspective

Closing this gap demands a multipronged, multimodal strategy. In the short term, clinicians should establish two complementary pathways for residual-risk assessment. The first pathway is an inherited-risk pathway based on once-in-a-lifetime Lp(a) measurement. An elevated Lp(a) concentration, commonly defined in clinical risk-enhancement frameworks as ≥125 nmol/L or ≥50 mg/dL, should prompt intensified LDL-C lowering and comprehensive management of blood pressure, diabetes, smoking, body weight, and other modifiable risk factors. Premature ASCVD among first-degree relatives should be documented, and cascade testing may be considered where clinically appropriate. The second pathway is an inflammatory-risk pathway based on hs-CRP measurement in clinically stable patients. An hs-CRP concentration ≥1.0 mg/L may be used as a locally proposed threshold for identifying residual inflammatory risk in Chinese patients with coronary artery disease, but this value should not be interpreted as a stand-alone treatment threshold. Acute infection, recent hospitalization, trauma, uncontrolled inflammatory disease, and other secondary causes should be considered, and repeat testing may be appropriate when the initial result is unexpected or potentially confounded. In appropriately selected secondary-prevention patients with persistent inflammatory risk, low-dose colchicine may be considered after assessment of renal and hepatic function, gastrointestinal tolerability, potential drug interactions, and patient preferences. Elevated Lp(a) may identify a patient with additional inherited atherogenic risk, but it should not independently determine colchicine initiation. Patients with concomitant elevation of Lp(a) and hs-CRP may represent a particularly important group for comprehensive risk-factor intensification and prospective study; however, this two-biomarker phenotype has not yet been validated as a colchicine-treatment algorithm. Table 1 summarizes the complementary biological roles, testing approaches, suggested interpretations in the Chinese context, immediate clinical actions, and important limitations of Lp(a), hs-CRP, their combination, and low-dose colchicine within a multimodal residual-risk framework. Aggressive lifestyle modification targeting central obesity, dietary patterns, and physical activity remains a synergistic cornerstone, as each of these factors independently modulates systemic inflammation.

**Table 1:** Complementary roles of Lp(a), hs-CRP, and low-dose colchicine in residual-risk management.

Component	Biological meaning	Typical testing approach	Suggested interpretation in China	Immediate clinical action	Important limitations
Lp(a)	Relatively stable, genetically determined atherogenic risk; associated with ASCVD and calcific aortic valve disease	Once-in-a-lifetime measurement in adulthood; repeat only in selected clinical situations	≥125 nmol/L or ≥50 mg/dL is commonly used as a risk-enhancing level; assay-specific interpretation is required	Intensify LDL-C lowering and comprehensive risk-factor control; assess premature ASCVD family history; consider cascade testing	mg/dL and nmol/L are not directly interchangeable; assay standardization remains important; elevated Lp(a) is not an indication for colchicine
hs-CRP	Dynamic marker of systemic inflammatory activity and residual inflammatory risk	Measure when clinically stable; repeat if unexpectedly elevated or potentially confounded	hs-CRP ≥1.0 mg/L may be used as a proposed Chinese risk-stratification threshold; ≥2 or ≥3 mg/L may be relevant in other studies	Exclude infection and secondary causes; optimize lifestyle and metabolic risk; consider anti-inflammatory therapy in selected secondary prevention	Affected by infection, obesity, diabetes, smoking, CKD, periodontal disease, and other conditions; threshold is not equivalent to a colchicine-treatment threshold
Lp(a) + hs-CRP	Combined inherited and dynamic residual-risk phenotype	Use both tests according to their different biological behavior	Concomitant elevation may identify a high-priority group for comprehensive prevention	Intensify global risk-factor management; consider specialist assessment; prioritize prospective research	The combined phenotype has not been validated as a treatment-allocation algorithm
Low-dose colchicine	Pharmacological reduction of inflammatory signaling in selected ASCVD patients	Not a biomarker test; treatment decision after clinical and safety assessment	May be considered in selected secondary-prevention patients with persistent inflammatory risk and guideline-directed therapy	Review renal/hepatic function, blood counts, gastrointestinal tolerance, and CYP3A4/P-glycoprotein interactions; monitor after initiation	Not indicated solely by elevated Lp(a); long-term safety, adherence, population selection, and China-specific effectiveness require further study
Standard ASCVD prevention	Reduction of modifiable atherothrombotic risk	Repeated clinical assessment and standard lipid/metabolic testing	Required regardless of Lp(a) or hs-CRP status	LDL-C/non-HDL-C control, BP control, diabetes treatment, smoking cessation, weight management, physical activity, and adherence support	Biomarkers should enhance—not replace—clinical risk assessment and guideline-directed therapy

ASCVD, atherosclerotic cardiovascular disease; hs-CRP, high-sensitivity C-reactive protein; Lp(a), lipoprotein(a). Thresholds should be interpreted in the context of the applicable guideline, assay characteristics, clinical setting, and overall ASCVD risk. Elevated Lp(a) should not independently determine colchicine initiation. The proposed combined Lp(a)-hs-CRP phenotypes require prospective validation before being used as treatment-allocation criteria.

The search for anti-inflammatory strategies need not be confined to colchicine. Intriguingly, a recent randomized, double-blind controlled trial of Xueshuantong Injection (a Panax notoginseng saponin preparation) demonstrated significantly greater reductions in CRP, interleukin-6, and matrix metalloproteinase-9 in patients with unstable angina, alongside improvements in clinical symptoms, with good safety and tolerability (27). While the sample size was modest and the endpoint surrogate, these findings open a window onto complementary anti-inflammatory approaches that merit rigorous investigation within China's integrative medicine framework.

In the medium to long term, the generation of China-specific evidence is paramount and non-negotiable. Pragmatic registry-based randomized trials or large-scale prospective cohort studies, embedded within the China Cardiovascular Disease Database or similar platforms, should evaluate low-dose colchicine in demographically and clinically representative populations, with deliberate stratification by diabetes status, renal function, and background pharmacotherapy. Such studies must incorporate pharmacogenomic sub-analyses to probe East Asian-specific polymorphisms that influence colchicine metabolism and inflammatory response, thereby laying the groundwork for precision anti-inflammatory therapy. Parallel prospective validation of hs-CRP thresholds—ideally through collaborative cohorts across East Asia—is essential to refine risk discrimination algorithms and avoid both over- and under-treatment. Health technology assessment and economic modeling, informed by local cost and utilization data, are equally urgent. While North American analyses have demonstrated that low-dose colchicine is highly cost-effective for secondary prevention, analogous studies calibrated to China's tiered healthcare economy and pricing structures are lacking (28). Such analyses would empower policymakers and insurance agencies to make evidence-informed coverage decisions.

Policy-level actions must enable and sustain these changes. National and provincial cardiovascular prevention guidelines should establish an integrated residual-risk module that includes: (1) once-in-a-lifetime Lp(a) measurement with standardized assay and reporting procedures; (2) context-specific hs-CRP measurement in clinically stable patients; (3) clear management pathways for intensifying LDL-C lowering and other modifiable risk factors when Lp(a) is elevated; (4) safety-based criteria for considering low-dose colchicine in selected secondary-prevention patients with persistent inflammatory risk; and (5) electronic health-record prompts and quality indicators to support implementation. Government investment is needed to expand access to Lp(a) and hs-CRP testing in county-level hospitals and community health centers, strengthen laboratory quality assurance, subsidize evidence-based secondary-prevention therapy, and fund continuing medical education for clinicians, pharmacists, and laboratory professionals. Implementation should be tiered: basic testing and risk-factor intensification can occur in primary and secondary hospitals, whereas complex drug-interaction assessment and colchicine initiation may be supported by regional cardiovascular prevention or lipid clinics.

From the authors' perspective, we are witnessing the definitive transition from a primarily lipid-centric “statin era” to an integrated “lipid-lowering plus anti-inflammatory” era in secondary prevention. For China, where the absolute cardiovascular event rate and the inflammatory burden are both extraordinarily high, the low cost and proven efficacy of colchicine represent a historically underleveraged public health opportunity. The imperative is not merely to adopt global recommendations wholesale, but to adapt them intelligently—calibrating thresholds, accumulating local evidence, building implementation capacity, and addressing safety concerns with rigorous pharmacovigilance. The path forward demands a coalition of researchers, frontline clinicians, professional societies, industry partners, and health authorities, all aligned around a shared commitment to sharply reduce the unacceptable burden of recurrent cardiovascular events through inflammation-informed care.

## Conclusion

Residual cardiovascular risk is biologically heterogeneous, clinically important, and only partially captured by conventional lipid profiles. The 2025 ACC recommendations highlight the relevance of inflammatory-risk assessment and targeted anti-inflammatory therapy in selected patients with established ASCVD, while the 2026 ACC/AHA multisociety dyslipidemia guideline provides a Class I recommendation for once-in-a-lifetime Lp(a) measurement. Together, these developments support a multimodal framework that distinguishes relatively stable inherited atherogenic risk from dynamic inflammatory risk.

For China, implementation should begin with practical and evidence-aligned steps: once-in-a-lifetime Lp(a) testing, context-specific hs-CRP measurement, aggressive control of LDL-C and other modifiable risk factors, and carefully selected use of low-dose colchicine in secondary-prevention patients with persistent inflammatory risk and acceptable safety profiles. Elevated Lp(a) should not be regarded as an independent indication for colchicine. Future work should prioritize assay standardization, China-specific outcome studies, pragmatic colchicine trials, family-based Lp(a) screening strategies, pharmacovigilance, and cost-effectiveness analyses. By integrating inherited-risk recognition, inflammatory-risk assessment, and evidence-based therapy without conflating their clinical roles, China can move toward a more precise, equitable, and implementable model of cardiovascular prevention.

## Data Availability

The original contributions presented in the study are included in the article/supplementary material, further inquiries can be directed to the corresponding author.
